# Non Electrocardiographic alterations in exercise testing in asymptomatic women. Associations with cardiovascular risk factors

**DOI:** 10.6061/clinics/2019/e1005

**Published:** 2019-09-10

**Authors:** Ricardo Quental Coutinho, Ulisses Ramos Montarroyos, Isly Maria Lucena de Barros, Maria José Bezerra Guimarães, Laura Olinda Bregieiro Fernandes Costa, Ana Kelley de Lima Medeiros, Maria de Fátima Monteiro, Moacir de Novaes Lima Ferreira, William Azem Chalela, Rodrigo Pedrosa

**Affiliations:** I Faculdade de Ciencias Medicas, Universidade de Pernambuco, Recife, PE, BR; II Programa de Doutorado em Ciencias da Saude, Instituto de Ciencias Biologicas, Faculdade de Ciencias Medicas, Universidade de Pernambuco, Recife, PE, BR; III Nucleo de Pos-Graduacao, Hospital Universitario Oswaldo Cruz, Universidade de Pernambuco, Recife, PE, BR; IV Centro de Estudos do Hospital Universitario Oswaldo Cruz, Universidade de Pernambuco, Recife, PE, BR; V Departamento Materno Infantil, Faculdade de Ciencias Medicas, Universidade de Pernambuco, Recife, PE, BR; VI Pronto Socorro Cardiologico de Pernambuco, Universidade de Pernambuco, Recife, PE, BR; VII Instituto do Coracao (InCor), Hospital das Clinicas HCFMUSP, Faculdade de Medicina, Universidade de Sao Paulo, Sao Paulo, SP, BR

**Keywords:** Exercise Test, Exercise Tolerance, Women, Middle-Aged

## Abstract

**OBJECTIVES:**

To estimate the prevalence of exercise testing alterations in middle-aged women without symptoms of heart disease and to verify the associations of functional capacity and heart rate behavior during and after exercise with cardiovascular risk factors.

**METHODS:**

A cross-sectional study was conducted with 509 asymptomatic women aged between 46 and 65 years who underwent clinical evaluations and exercise testing (Bruce protocol). The heart rate behavior was evaluated by the maximal predicted heart rate achieved, chronotropic index and recovery heart rate.

**RESULTS:**

The mean age was 56.4±4.8 years, and 13.4% of the patients had a Framingham risk score above 10%. In the exercise treadmill testing, 58.0% presented one or more of the following alterations (listed in order of ascending prevalence): symptoms (angina, dyspnea, and dizziness), ST-segment depression, arrhythmia, reduction in recovery heart rate of ≤12 bpm at 1 minute, altered maximal predicted heart rate achieved, abnormal blood pressure, functional capacity deficiency, and altered chronotropic index. In the multivariate analysis, the following associations (odds ratio) were observed for these alterations: chronotropic index was associated with obesity (2.08) and smoking (4.47); maximal predicted heart rate achieved was associated with smoking (6.45); reduction in the recovery heart rate at 1 minute was associated with age (1.09) and obesity (2.78); functional capacity was associated with age (0.92), an overweight status (2.29) and obesity (6.51).

**CONCLUSIONS:**

More than half of middle-aged women without cardiovascular symptoms present alterations in one or more exercise testing parameters. Alterations in the functional capacity or heart rate behavior, as verified by exercise testing, are associated with age, smoking, an overweight status and obesity.

## INTRODUCTION

Exercise testing has been used for the prognostic evaluation of general and cardiovascular mortality and following the occurrence of nonfatal cardiac events 1-5. Among the parameters of exercise testing, ventricular ectopic beats in recovery and nonelectrocardiographic parameters, including functional capacity, maximal predicted heart rate (HR) achieved (MPHRA) and HR, are considered predictors of cardiovascular mortality 3-6.

The relevance of functional capacity as a predictor of fatal cardiovascular events was verified in cohort studies performed exclusively with the female sex 7-9. Regarding the HR behavior in the exercise testing, an abnormal response at peak or at recovery is also considered a predictor of death, regardless of the Framingham risk score 7,10,11. These findings are highlighted in the guidelines for exercise testing from various scientific societies 1,2-6,12-14.

In asymptomatic women, the prognostic value of these nonelectrocardiographic parameters of exercise testing 4,7,8,15 may support their use in clinical practice and in primary prevention programs since they add value to traditional cardiovascular risk scores. This contribution is particularly relevant for middle-aged women due to an important change in the cardiovascular risk profile related to estrogen deficiency and due to the high prevalence of smoking, obesity, dyslipidemias, diabetes and hypertension 16,17.

However, the prevalence of alterations in the nonelectrocardiographic parameters of exercise testing that serve as predictors of mortality and their associations with cardiovascular risk factors are still poorly studied in middle-aged women. In general, studies 7-11,15 that investigate the association of functional capacity and HR behavior during and after exercise with other variables use fatal cardiovascular events as the outcome. Moreover, the parameters of exercise testing that can serve as predictors of cardiovascular events are not always explicit in the results of the examination and are therefore not used in clinical practice. Thus, the present study aims to estimate the prevalence of alterations in exercise testing in middle-aged asymptomatic women and to verify the association of functional capacity and HR behavior with traditional cardiovascular risk factors.

## METHODS

A cross-sectional study was carried out in middle-aged women, and this project was approved by the Ethics Committee of the institution where the data were collected (CAAE 0159.0.106.106-11). Participants signed a Free and Informed Consent Term form, and specialized follow-up was offered to women who presented significant alterations in the exercise testing, such as limiting angina, ischemic depression and complex arrhythmia.

### Sample and data collection

A total of 509 women who were aged 46-65 years and did not have cardiovascular symptoms were selected from two outpatient services for women. The exclusion criteria included the following: a history of referred or clinically evident cardiovascular disease, except systemic arterial hypertension; a diagnosis of liver or kidney disease; the use of corticosteroids or hormone replacement therapy; pregnancy; the use of an intrauterine device; the use of hormonal contraceptives in the past year; and contraindications to exercise testing according to the III Guideline on Exercise Testing of the Brazilian Society of Cardiology 1.

A structured questionnaire was used to evaluate variables related to cardiovascular risk factors, which included the woman's history, the use of specific medications, clinical examinations and biochemical tests. The body mass index (BMI=weight/height^2^) was used to classify women into three categories: eutrophic (<24.9 kg/m^2^), overweight (25-29.9 kg/m^2^) and obese (>30 kg/m^2^). Exercise testing was performed by the principal investigator following the Bruce protocol 18 (Inbramed® treadmill with the ErgoPc® exercise testing program and a Unitec® mercury column tensiometer); testing was discontinued by a threshold symptom criterion, which was generally muscle and/or respiratory fatigue. The interpretation of the results of the exercise testing were based on the parameters established by the Brazilian Society of Cardiology 1.

To examine the variables of the exercise testing considered predictors of mortality 3,4,15, the following formulas were used: (a) functional capacity = (maximum VO^2^ reached x 100)/VO^2^ predicted, where the predicted VO^2^ = 14.7 - (0.13 x age); (b) MPHRA = peak HR x 100/predicted HR; (c) chronotropic index = (peak HR - resting HR) x 100/(predicted HR - resting HR). To estimate the MPHRA and chronotropic index, the predicted HR = (220 - age), as recommended by Karnoven et al. 19; and (d) reduction in recovery HR at 1 minute = peak HR - recovery HR at 1 minute. The HR behavior was evaluated by the MPHRA, chronotropic index and recovery HR. According to previous reports 3,4,13, the cutoff value of functional capacity was considered to be less than 85%; the cutoff for the reduction in recovery HR at 1 minute was equal to or less than 12 bpm; the cutoff for the MPHRA was less than 85%; and the cutoff for chronotropic index was below 80%. For the last two variables, in women taking HR-lowering medication, values ??below 62% were classified as altered 3,4,6.

### Statistical analysis

For continuous variables, the data is described by the mean and dispersion measurements (standard deviation and the minimum and maximum values). The normality of the distribution was verified by the Komogorov-Smirnov test. For categorical variables, the absolute and relative frequency distributions are shown. The prevalence of alterations in the variables evaluated in the exercise testing was calculated, and 95% confidence intervals (CIs) are presented.

In the analysis of the associations between functional capacity and HR behavior with cardiovascular risk factors, the association measures of the odds ratio (OR) and the 95% CI were calculated. In the final modeling, a multivariate logistic regression technique with a stepwise method was used that included variables that were statistically significant in the bivariate analysis (below 20%; *p*<0.2). Statistically significant variables (below 10%; *p*<0.1) were maintained in the model. A database was built in the Microsoft Office Access program, and data were analyzed in the Stata program (version 12.0).

## RESULTS

The study sample, as shown in Table 1, consisted of 509 women with a mean age of 56.4±4.8 years, of whom 11.2% were diabetic patients and 13.4% had a Framingham risk score above 10%. This table also includes the descriptive measures of exercise testing variables, including peak HR and recovery HR at 1 minute, including the mean when appropriate. In only 15 (3.1%) women, the exercise testing was interrupted before the women reached fatigue, most frequently for reasons unrelated to the cardiovascular system (vestibular syndrome, orthopedic limitations, and treadmill maladaptation). The exercise was interrupted in three women due to limiting chest pain and dyspnea that were compatible with myocardial ischemia. One of the patients had ischemic ST changes associated with ventricular tachycardia and underwent cardiac catheterization and percutaneous coronary intervention.

The prevalence of alterations in exercise testing (Table 2) varied between 2.2% (the presence of symptoms: angina, dyspnea, and dizziness) and 27.7% (altered chronotropic index). Of the 22 (4.3% of all women) patients with ST depression, eleven (2.2%) presented a depression equal to or greater than one millimeter, with a horizontal or descending pattern. These women were referred for specialized follow-ups. Alterations in one or more variables were observed in 27.9% of the women, who presented with symptoms, arrhythmia, ST-segment depression and/or abnormal blood pressure. On the other hand, 44.0% presented alterations in one or more of the four nonelectrocardiographic variables predicting mortality. One or more alterations in these eight parameters were identified in 58.0% of the women.

In the bivariate analysis (Table 3), the following associations between alterations in the nonelectrocardiographic variables predicting mortality and cardiovascular risk factors were identified: (a) chronotropic index was associated with obesity, hypertension and smoking; (b) MPHRA was associated with smoking; (c) reduction in recovery HR at 1 minute was associated with age, BMI, obesity, hypertension and three or four cardiovascular risk factors; and (d) functional capacity was associated with age, age from 55-60 years and 61-65 years, BMI, an overweight status and obesity.

In the multivariate analysis (Table 4), the following associations remained: chronotropic index with obesity (OR=2.08) and smoking (OR=4.47); altered MPHRA with smoking (OR=6.45); mild reduction in recovery HR at 1 minute with age (OR=1.09) and obesity (OR=2.78); alteration in functional capacity with age (OR=0.92), an overweight status (OR=2.29) and obesity (OR=6.51). Obesity was associated with the highest number of predictive variables that were altered in the exercise testing and, together with smoking, presented the highest risk.

## DISCUSSION

The present study found that more than half of the middle-aged women in the sample, who had no previous cardiovascular symptoms, had one or more altered parameters in exercise testing. The presence of symptoms and the depression of the ST segment occurred less frequently, and alterations in the nonelectrocardiographic variables predicting mortality, especially chronotropic index and functional capacity, were observed more frequently. The study also showed that alterations in functional capacity or HR behavior in exercise testing are associated with age, smoking, an overweight status and obesity.

The prevalence of alterations in exercise testing variables observed in this study differs from those in asymptomatic women with a similar mean age that were described by some authors 8,15,20-22. Here, a lower prevalence was observed than that described by other authors for ST-segment depression 8,20-22, reduction in recovery HR of ≤12 bpm at 1 minute 22 and the proportion of functional capacity attained in relation to the estimated value for functional capacity <85% 15. On the other hand, the prevalence of MPHRA <85% 20 and chronotropic index <80% was higher here than in other studies 15,20.

Demographic characteristics, cardiovascular risk factors, reasons for the interruption of the exam and formulas used to calculate the predictors of mortality should be taken into account when comparing the prevalence of exercise testing alterations 11,21,23. In this study, exercise testing interruptions were symptom-limited. The interruption by estimated HR without fatigue results in a higher prevalence of altered functional capacity. Regarding the formulas used to determine the MPHRA and chronotropic index, the equation that is traditionally used (= 220 - age) 19 was used to calculate the maximum estimated HR; however, some studies used other calculation methods 11,20,21, which resulted in a different prevalence of alterations for these predictive variables. In this regard, in middle-aged women, Gulati et al. 1 observed that the prevalence of an MPHRA <85% and a chronotropic index <80% that were observed when using the traditional calculation of the estimated HR were, respectively, 2.3 and 1.9 times higher than those obtained from another formula [= 206 - (0.88 x age)].

Regarding the associations that were studied, the use of functional capacity and the HR response as the main outcomes are supported. In general, these nonelectrocardiographic variables of exercise testing are used in multivariate analyses as independent variables to examine the associations with acute myocardial infarction and death 7-11,21,22,24,25. Nevertheless, in the initial stage of some studies 11,24,25 that verified the predictive value of the nonelectrocardiographic variables of the exercise testing for mortality, the bivariate associations between functional capacity, chronotropic response, chronotropic index and reduction in recovery HR at 1 minute with age, sex, BMI, systemic arterial hypertension, smoking, dyslipidemias, diabetes and other cardiovascular risk factors were evaluated.

The bivariate associations of the MPHRA with smoking and a reduction in the recovery HR of ≤12 at 1 minute with age and hypertension that were observed in this study were also identified by Maddox et al. 24. However, unlike the present study, these authors 24 also observed bivariate associations of these variables with diabetes and dyslipidemia. Bivariate associations between a chronotropic index of less than 80% and obesity, hypertension and smoking were observed in this study and were also found in a cohort of st. James 11. More recently, similar to the findings of the present study, Abdelmoneim et al. 20 did not observe a bivariate association of chronotropic index with diabetes and dyslipidemia. Regarding functional capacity <85%, in agreement with Peterson et al. 26, a bivariate association of this variable with age and obesity was identified. However, in this study, there were no associations with smoking, diabetes and hypertension, as verified by other authors 26. The multivariate analysis highlighted that obesity represented the main risk association for low functional capacity and a mild reduction in recovery HR at 1 minute in the present study. Smoking was the main factor associated with the MPHRA and chronotropic index. Therefore, the significant association of the variables of exercise testing predicting mortality with obesity and smoking should be emphasized. These factors, together with low functional capacity, can be modified by preventive actions.

The discrete protective association of advancing age for functional capacity was an unexpected finding, and it is possible that other variables that were not tested, such as regular physical activity and the specialized follow-up for the women of the sample with greater age, influenced this result. A limitation of this study was that because it was a cross-sectional study, it is important to test the associations that were observed in a longitudinal study. In addition, the sample studied was drawn from specialized public services, which restricts the extrapolation of these results to the general population.

In this study, the sample was composed of middle-aged women who have less representation in this type of study and in large clinical trials worldwide 27. In clinical practice, there are fewer requests for exercise testing for middle-aged women, suggesting that the minimum parameters are not optimized and that there is a high prevalence of false positives in this group 28. In the present study, an adequate number of participants achieved the average parameters for exercise time and maximum HR, and there was a low prevalence of ST alterations, which allowed the appropriate interpretation of this test.

It should be emphasized that the exercise testing variables related to functional capacity and the HR behavior at the peak and after the peak, which were included in the present study, are not always valued in the clinical interpretation of exercise testing despite their importance as predictors of mortality. Supporting the importance of these factors, a large trial 29 with low-risk women with suspected coronary artery disease showed no difference in exercise testing with or without myocardial scintigraphy in the prediction of two-year survival. In addition, exercise testing alone was more cost-effective. The lack of exposure to radiation and the logistic ease of exercise testing should be considered, especially in countries such as Brazil, with technological inequalities and limited health resources.

The present study supports the use of exercise testing in middle-aged women by using a symptom-limited Bruce protocol, with the evaluation of variables considered predictors of mortality, to identify women who need follow-up with other investigations. Therefore, these findings support exercise testing in the routine investigation of middle-aged women, as well as the inclusion of this test in programs for the prevention of cardiovascular events and health promotion.

In summary, one or more alterations in exercise testing related to functional capacity, HR behavior, the presence of symptoms, and electrocardiographic and blood pressure abnormalities were observed in more than half of the tested middle-aged women who are had no cardiovascular symptoms and no known heart disease. Alterations in functional capacity or HR behavior, as verified by exercise testing, are associated with age, smoking, an overweight status and obesity.

## AUTHOR CONTRIBUTIONS

Coutinho RQ was responsible for planning and supervising the research, collecting data, discussing the results, writing the manuscript, making the final manuscript revisions and submitting the manuscript. Montarroyos UR was responsible for planning the research, performing statistical analysis, discussing the results, and writing and critically revising the manuscript. Barros IML was responsible for planning and supervising the research, collecting data and critically revising the manuscript. Guimarães MJB was responsible for planning the research, discussing the results, writing the manuscript, making the final manuscript revisions and submitting the manuscript. Costa LOBF was responsible for planning the research. Medeiros AKL and Monteiro MF were responsible for collecting data. Ferreira MNL and Chalela WA were responsible for planning the research and critically revising the manuscript. Pedrosa R was responsible for planning the research, discussing the results, and writing and critically revising the manuscript. All authors have read and approved the final version of the manuscript.

## Figures and Tables

**Table 1:** Baseline characteristics of the sample and the exercise testing.

Characteristics	Statistics (n = 509)
**Age **(years)*	56.4±4.8 (46-65)
**BMI **(kg/m^2^)*	27.8±4.9 (14.8-42.1)
**Age group **(years) (n,%)	
46-50	62 (12.2%)
51-55	175 (34.4%)
56-60	149 (29.3%)
61-65	123 (24.2%)
**Clinical history **(n, %)	
Diabetes	57 (11.2%)
Systemic arterial hypertension	247 (48.5%)
Dyslipidemia	211 (41.5%)
Obesity	158 (31.0%)
Smoking	39 (7.7%)
Use of β-blockers	57 (11.2%)
**Number of cardiovascular risk factors^†^ (n, %)**	
None	125 (24.6%)
One	157 (30.8%)
Two	138 (27.1%)
Three or more	89 (17.5%)
**Framingham risk score^‡^ (n,%)**	
<5%	409 (80.3%)
5 to 10%	32 (6.3%)
>10%	68 (13.4%)
Exercise testing*	
Resting HR (bpm)	74.4±13.3 (37-136)
Resting SBP (mm Hg)	138.4±18.1 (90.0-210.0)
Resting DBP (mm Hg)	85.1±8.9 (50.0-125.0)
Peak HR (bpm)	152.4±19.6 (79-200)
Recovery HR at 1 minute (bpm)	130.7±19.3 (69-183)
Functional capacity (VO^2^/METs)	7.6±2.0 (1.5-14.7)
MPHRA	93.2±11.5 (50.3-122.7)
Chronotropic index	88.4±20.8 (17.0-155.6)
Reduction in recovery HR at 1 minute	21.8±9.1 (-2-76)
Percent of the functional capacity reached relative to the estimated functional capacity	104.9±26.9 (23.8-230.5)

BMI – Body mass index; HR – Heart rate; SBP – Systolic blood pressure; DBP – Diastolic blood pressure; MPHRA – Maximal predicted heart rate achieved.

* Average ± standard deviation (minimum value - maximum value); ^†^Cardiovascular risk factors: diabetes, systemic arterial hypertension, dyslipidemia, obesity and smoking; ^‡^Women with diabetes were classified as having a Framingham risk score >10%.

**Table 2:** Prevalence of alterations in the exercise testing variables in middle-aged asymptomatic women.

Exercise testing	Prevalence	
	N (%)	95% CI
**Parameters evaluated**		
Symptoms (angina, dyspnea, and dizziness)	11 (2.2%)	1.2%-3.8%
Abnormal blood pressure	93 (18.3%)	15.2%-21.9%
Arrhythmia	43 (8.4%)	6.3%-11.2%
ST depression	22 (4.3%)	2.9%-6.5%
MPHRA<85%*	73 (14.3%)	11.2%-17.2%
Chronotropic index <80%	141 (27.7%)	23.8%-31.5%
Reduction in recovery HR at 1 minute ≤12 bpm	69 (13.6%)	10.8%-16.8%
Percent of functional capacity reached relative to the estimated <85% functional capacity	118 (23.2%)	19.5%-26.9%
**Change in one or more parameters**		
Symptoms, arrhythmia and/or ST depression	67 (13.2%)	10.5%-16.4%
Symptoms, arrhythmia, ST depression and/or abnormal blood pressure	142 (27.9%)	24.2%-32.0%
MPHRA <85%, chronotropic index <80%, reduction in recovery HR of ≤12 bpm at 1 minute and/or altered functional capacity	224 (44.0%)	39.9%-48.6%
One or more of the eight evaluated parameters altered	295 (58.0%)	53.6%-62.2%

MPHRA – Maximal predicted heart rate achieved; HR – Heart rate.

* MPHRA and chronotropic index <62% were considered altered in women (n=57) using β-blockers.

**Table 3:** Bivariate analysis of the alterations in the predictive variables of exercise testing with cardiovascular risk factors in middle-aged asymptomatic women.

Variables	Chronotropic index			MPHRA			Reduction in recovery HR at 1 minute			Percent of functional capacity		
	Altered	OR (95% CI)	*p*-value	Altered	OR (95% CI)	*p*-value	Altered	OR (95% CI)	*p*-value	Altered	OR (95% CI)	*p*-value
**Age **(years)	56.6±5.2	1.01 (0.97-1.06)	0.482	57.0±5.3	1.03 (0.98-1.09)	0.238	58.0±5.1	1.09 (1.03-1.15)	0.002	55.1±4.9	0.93 (0.89-0.97)	0.001
**Age group** (years)												
46-50	20 (32.3%)	1.00	-	10 (16.1%)	1.00	-	8 (12.9%)	1.00	-	23 (37.1%)	1.00	-
51-55	45 (25.7%)	0.73 (0.39-1.37)	0.322	22 (12.6%)	0.74 (0.33-1.68)	0.482	16 (9.1%)	0.68 (0.27-1.68)	0.401	47 (26.9%)	0.62 (0.33-1.15)	0.131
56-60	32 (22.1%)	0.59 (0.31-1.15)	0.125	16 (10.7%)	0.63 (0.27-1.47)	0.281	15 (10.1%)	0.76 (0.31-1.90)	0.559	26 (17.4%)	0.36 (0.18-0.70)	0.003
61-65	43 (35.2%)	1.14 (0.60-2.19)	0.687	25 (20.3%)	1.33 (0.59-2.97)	0.492	30 (24.4%)	2.17 (0.93-5.09)	0.072	22 (18.6%)	0.37 (0.19-0.74)	0.005
**BMI**												
In kg/m^2^	28.3±5.2	1.03 (0.99-1.07)	0.113	28.0±5.2	1.01 (0.96-1.06)	0.773	29.1±5.3	1.06 (1.01-1.11)	0.020	30.7±5.0	1.17 (1.12-1.23)	<0.001
Eutrophic	39 (24.7%)	1.00	-	21 (13.3%)	1.00	-	12 (7.6%)	1.00	-	16 (10.1%)	1.00	-
Overweight	46 (23.7%)	0.95 (0.58-1.55)	0.832	25 (12.9%)	0.97 (0.52-1.80)	0.911	28 (14.4%)	2.03 (1.00-4.15)	0.050	37 (19.1%)	2.09 (1.11-3.92)	0.021
Obese	56 (35.9%)	1.66 (1.02-2.71)	0.042	27 (17.2%)	1.35 (0.73-2.51)	0.336	29 (18.5%)	2.73 (1.34-5.59)	0.006	65 (41.4%)	6.27 (3.42-11.5)	<0.001
**Clinical history**												
SAH	80 (32.5%)	1.59 (1.07-2.34)	0.021	32 (13.0%)	0.80 (0.49-1.32)	0.387	45 (18.3%)	2.22 (1.31-3.77)	0.003	65 (26.4%)	1.41 (0.93-2.13)	0.105
Diabetes	17 (29.8%)	1.12 (0.61-2.05)	0.711	9 (15.8%)	1.14 (0.53-2.43)	0.741	10 (17.5%)	1.41 (0.68-2.95)	0.356	18 (31.6%)	1.62 (0.89-2.96)	0.114
Dyslipidemia	53 (25.2%)	0.80 (0.54-1.19)	0.277	24 (11.4%)	0.65 (0.38-1.10)	0.107	33 (15.6%)	1.34 (0.80-2.23)	0.261	42 (19.9%)	0.72 (0.47-1.11)	0.136
Smoking	22 (56.4%)	3.80 (1.95-7.39)	<0.001	18 (46.1%)	6.45 (3.24-12.8)	<0.001	2 (5.1%)	0.32 (0.08-1.37)	0.126	10 (25.6%)	1.15 (0.54-2.44)	0.711
Number of cardiovascular risk factors*												
None	29 (23.2%)	1.00	-	17 (13.6%)	1.00	-	11 (8.8%)	1.00	-	27 (21.6%)	1.00	-
One	37 (23.6%)	1.02 (0.59-1.77)	0.942	20 (12.7%)	0.93 (0.46-1.86)	0.832	22 (14.1%)	1.70 (0.79-3.66)	0.174	28 (17.8%)	0.79 (0.44-1.42)	0.428
Two	47 (34.1%)	1.71 (0.99-2.95)	0.054	21 (15.2%)	1.14 (0.57-2.27)	0.710	18 (13.0%)	1.55 (0.70-3.43)	0.347	35 (25.5%)	1.23 (0.70-2.19)	0.473
Three or four	27 (30.7%)	1.47 (0.79-2.71)	0.223	13 (14.6%)	1.09 (0.50-2.37)	0.834	19 (21.4%)	2.81 (1.26-6.26)	0.011	28 (31.8%)	1.67 (0.90-3.09)	0.105

MPHRA – Maximal predicted heart rate achieved; HR – Heart rate; BMI – Body mass index; SAH – Systemic arterial hypertension.

* Cardiovascular risk factors: SAH, diabetes. dyslipidemia and smoking.

**Table 4:** Multivariate analysis of alterations in the predictive variables of exercise testing with cardiovascular risk factors in middle-aged asymptomatic women.

Variables	Alterations in exercise testing							
	Chronotropic index		MPHRA		Reduction in recovery HR at 1 minute		Percent of functional capacity	
	OR (95% CI)	*p*-value	OR (95% CI)	*p*-value	OR (95% CI)	*p*-value	OR (95% CI)	*p*-value
Age (years)					1.09 (1.03-1.15)	0.003	0.92 (0.88-0.97)	0.001
Eutrophic	1.0	-			1.0	-	1.0	-
Overweight	1.10 (0.66-1.83)	0.717			1.89 (0.92-3.87)	0.083	2.29 (1.21-4.33)	0.011
Obese	2.08 (1.24-3.47)	0.005			2.78 (1.35-5.71)	0.005	6.51 (3.52-12.0)	<0.001
Smoking	4.47 (2.24-8.90)	<0.001	6.45 (3.24-12.8)	<0.001				

MPHRA – Maximal predicted heart rate achieved; HR – Heart rate.
